# Kernel-based joint independence tests for multivariate stationary and non-stationary time series

**DOI:** 10.1098/rsos.230857

**Published:** 2023-11-29

**Authors:** Zhaolu Liu, Robert L. Peach, Felix Laumann, Sara Vallejo Mengod, Mauricio Barahona

**Affiliations:** ^1^ Department of Mathematics, Imperial College London, London SW7 2AZ, UK; ^2^ Department of Brain Sciences, Imperial College London, London W12 0NN, UK; ^3^ Department of Neurology, University Hospital Würzburg, Würzburg 97070, Germany

**Keywords:** multivariate time-series analysis, joint independence test, higher-order interaction, kernel method

## Abstract

Multivariate time-series data that capture the temporal evolution of interconnected systems are ubiquitous in diverse areas. Understanding the complex relationships and potential dependencies among co-observed variables is crucial for the accurate statistical modelling and analysis of such systems. Here, we introduce kernel-based statistical tests of joint independence in multivariate time series by extending the *d*-variable Hilbert–Schmidt independence criterion to encompass both stationary and non-stationary processes, thus allowing broader real-world applications. By leveraging resampling techniques tailored for both single- and multiple-realization time series, we show how the method robustly uncovers significant higher-order dependencies in synthetic examples, including frequency mixing data and logic gates, as well as real-world climate, neuroscience and socio-economic data. Our method adds to the mathematical toolbox for the analysis of multivariate time series and can aid in uncovering high-order interactions in data.

## 1. Introduction

Time series that record temporal changes in sets of system variables are ubiquitous across many scientific disciplines [1], from physics and engineering [2] to biomedicine [3,4], climate science [5,6], economics [7,8] and online human behaviour [9,10]. Many real-world systems are thus described as multivariate time series of (possibly) interlinked processes tracking the temporal evolution (deterministic or random) of groups of observables of interest. The relationships between the measured variables are often complex, in many cases displaying interdependencies among each other. For example, the spreading of COVID-19 in Indonesia was dependent on weather conditions [11]; the sustainable development goals (SDGs) have extensive interlinkages [12]; there are strong interconnections between foreign exchange and cryptocurrencies [13]; and the brain displays multiple spatial and temporal scales of functional connectivity [14]. Driven by technological advances (e.g. imaging techniques in the brain sciences [15], or the increased connectivity of personal devices via the Internet of Things [16]), there is a rapid expansion in the collection and storage of multivariate time-series datasets, which underlines the need for mathematical tools to analyse the interdependencies within complex high-dimensional time-series data.

Characterizing the relationships between variables in a multivariate dataset often underpins the subsequent application of statistical and machine-learning methods. In particular, before further analyses can be performed, it is often crucial to determine whether the variables of interest are jointly independent [17]. Joint independence of a set of *d* variables means that no subset of the *d* variables are dependent. We need to look no further than ANOVA and *t*-tests to find classic statistical methods that assume joint independence of input variables, and the violation of this assumption can lead to incorrect conclusions [18]. Causal discovery methods, such as structural equation modelling, also require joint independence of noise variables [19]. Furthermore, joint independence has applications in uncovering higher-order networks, an emergent area highlighted in recent studies [20–24].

Kernel-based methods offer a promising framework for testing statistical independence. Notably, the *d*-variable Hilbert–Schmidt independence criterion (dHSIC) [19] can be used as a statistic to test the joint independence of *d* random variables. Developed as an extension of the pairwise HSIC [25], a statistical test that measures the dependence between two variables [25–27], dHSIC measures the dependence between *d* variables [19]. Specifically, dHSIC can be simply defined as the ‘squared distance’ between the joint distribution and the product of univariate marginals when they are embedded in a reproducing kernel Hilbert space (RKHS). Crucially, kernel methods do not make assumptions about the underlying distributions or type of dependencies (i.e. they are non-parametric). Yet, in its original form, dHSIC assumes the data to be *iid* (i.e. drawn from identical independent distributions). This is an unreasonable assumption in the case of time-series data, and it has precluded its application to temporal data.

To the best of our knowledge, dHSIC has not yet been extended to time-series data. The pairwise HSIC has been extended to deal with *stationary* random processes under two different test resampling strategies: shifting within time series [26] and the Wild Bootstrap method [27]. However, the assumption of stationarity, by which the statistical properties (e.g. mean, variance, autocorrelation) of the time series are assumed not to change over time, is severely restrictive in many real-world scenarios, as non-stationary processes are prevalent in many areas, e.g. stock prices under regime changes or weather data affected by seasonality or long-term trends. Hence, there is a need for independence tests that apply to both stationary and non-stationary processes. Recently, pairwise HSIC has been extended to non-stationary random processes by using random permutations over independent realizations of each time series, when available [28].

In this paper, we show how dHSIC can be applied to reject joint independence in the case of both stationary and non-stationary multivariate random processes. Following recent work [28], we adapt dHSIC so that it can be applied to stationary and non-stationary time-series data when multiple realizations are present. Additionally, we develop a new bootstrap method inspired by [26], which uses ‘shifting’ to deal with stationary time-series data when only one realization is available. Using these methodological advances, we then introduce statistical tests that rely on these two different resampling methods to generate appropriate null distributions: one for single-realization time series, which is only applicable to stationary random processes, and another for multiple realization time series, which is applicable to both stationary and non-stationary random processes. We show numerically that the proposed statistical tests based on dHSIC robustly and efficiently identify the lack of joint independence in synthetic examples with known ground truths. We further show how recursive testing from pairwise to *d*-order joint independence can reveal emergent higher-order dependencies in real-world socio-economic time series that cannot be explained by lower-order factorizations.

## 2. Preliminaries

### 2.1. Kernel-based tests for joint independence

**Definition (Joint independence of a set of variables).**

The *d* variables *X*^*j*^, *j* = 1, …, *d*, with joint distribution $P_{X^{1},\ldots ,X^{d}}$ are jointly independent if and only if the joint distribution is fully factorizable into the product of its univariate marginals, i.e. $P_{X^{1},\ldots ,X^{d}}=\prod_{\;j=1}^{d}P_{X^{j}}$, where the $P_{X^{j}}$ denote the marginals.

**Remark (Joint independence of subsets).**

If *d* variables are jointly independent, then any subset of those *d* variables is also jointly independent, e.g. $P_{X^{1},X^{2},X^{3}}=P_{X^{1}}P_{X^{2}}P_{X^{3}}$ implies $P_{X^{1},X^{2}}=P_{X^{1}}P_{X^{2}}$, which follows from marginalization with respect to *X*^3^ on both sides of the equality. Hence, by the contrapositive, lack of joint independence of a subset of variables implies lack of joint independence of the full set of variables.

A series of papers in the last two decades have shown how kernel methods can be used to test for independence of random variables (for details, see [19,25]). The key idea is to embed probability distributions in RKHSs [29] via characteristic kernels, thus mapping distributions uniquely to points in a vector space. For a summary of the key definitions and foundational results, see [30,31].

**Definition (RKHS and mean embedding for probability distributions [32,33]).**

Let $H_{k}$ be a RKHS of functions f : X→R endowed with dot product 〈 · , · 〉, and with a reproducing kernel k : X×X→R. Let P be a distribution defined on a measurable space X, then the mean embedding of P in $H_{k}$ is an element $\mu_{P}\in H_{k}$ given by $\mu_{P}\;:=\int k(x,\cdot )\;P(dx)$, with the property $\langle f,\mu_{P}\rangle =E_{P}[f]=$ $\int f\;(x)P(dx),\;\forall f\in H_{k}$.

If the kernel is characteristic, the RKHS mapping is injective and this representation uniquely captures the information about each distribution. Based on such a mapping, statistics have been constructed to test for homogeneity (using the maximum mean discrepancy, MMD [33]) or independence (using the HSIC [25]) between two random variables.

**Remark.**

An example of a characteristic kernel is the Gaussian kernel $k_{\sigma }(x,y)=\exp\left(-\|x-y{\|}^{2}/\sigma^{2}\right)$ where $x,y\in R^{p}$. The Gaussian kernel will be used throughout our applications below, but our results apply to any other characteristic kernel.

Recently, an extension of HSIC for *d* variables, denoted dHSIC, was introduced and used as a statistic for joint independence to test the null hypothesis $H_{0}\;:\;P_{X^{1},\ldots ,X^{d}}=\prod_{\;j=1}^{d}P_{X^{j}}$.

**Definition (dHSIC [19]).**

Let us consider *d* random variables *X*^*j*^, *j* = 1, …, *d*, with joint distribution $P_{X^{1},\ldots ,X^{d}}$. For each *X*^*j*^, let $H_{k^{\;j}}$ denote a separable RKHS with characteristic kernel *k*^*j*^. The *d*-variable dHSIC, which measures the similarity between the joint distribution and the product of the marginals, is defined as

2.1 $$\text{dHSIC}(X^{1},\ldots ,X^{d})\;:=\|\mu_{P_{X^{1},\ldots ,X^{d}}}-\mu_{P_{X^{1}}\cdots P_{X^{d}}}{\|}_{H}^{2},$$

where $H\;:=H_{k^{1}}⊗\cdots ⊗H_{k^{d}}$ and ⊗ is the tensor product.

**Remark.**

Given the definition (2.1), dHSIC is zero if and only if the variables are jointly independent, i.e. when the joint distribution is equal to the product of the marginals. This is the basis for using dHSIC to define the null hypothesis for statistical tests of joint independence.

**Remark (Emergent high-order dependencies).**

As noted above, the rejection of joint independence for any subset of a set of *d* variables also implies the rejection of joint independence for the full set of *d* variables. Therefore, many observed rejections of joint independence at higher orders follow from rejections of joint independence at lower orders (i.e. within subsets of variables). To identify more meaningful high-order interactions, in some cases, we will also consider ‘first time rejections’ of *d*-way joint independence, i.e. when the joint independence of a set of *d* variables is rejected but the joint independence of each and all of its subsets of size *d*′ < *d* cannot be rejected. We denote these as emergent high-order dependencies.

### 2.2. Time series as finite samples of stochastic processes

Our interest here is in the joint independence of time series, which we will view as finite samples of stochastic processes.

**Notation (Stochastic processes and sample paths).**

We will consider a set of *d* stochastic processes $\{X^{j}(t;\omega )\;:\;t\in T\},\;j=1,\ldots ,d$, where t∈T is defined over the index set, corresponding to time, and ω∈Ω is defined over the sample space. Below, we will also use the shorthand $\left\{X_{t}^{j}\right\}$ to denote each stochastic process.

For each stochastic process, we may observe *n* independent realizations (or paths), which are samples from Ω indexed by *ω*_*i*_: $\{X^{j}(t;\omega_{i})\;:\;t\in T\},\;i=1,\ldots ,n$. Furthermore, each path is finite and sampled at times *t* = 1, …, *T*_*j*_.

**Remark (Time series as data samples).**

For each variable *X*^*j*^, the data samples (time series) consist of *n* paths (*X*^*j*^(1,*ω*_*i*_), …, *X*^*j*^(*T*_*j*_,*ω*_*i*_)), *i* = 1, …,*n*, which we arrange as *T*_*j*_-dimensional vectors $x_{i}^{j}=(x_{i,1}^{j},\ldots ,x_{i,T_{j}}^{j}),\;i=1,\ldots ,n$, i.e. the components of the vector are given by $x_{i,t_{k}}^{j}\;:\;=X^{j}(t_{k};\omega_{i})$.

**Definition (Independence of stochastic processes).**

Two stochastic processes $\left\{X_{t}^{j}\right\}$ and $\left\{X_{t}^{\;j^{\prime }}\right\}$ with the same index set T are independent if for every choice of sampling times $t_{1},\ldots ,t_{f}\in T$, the random vectors (*X*^*j*^(*t*_1_), …,*X*^*j*^(*t*_*f*_)) and (*X*^*j*′^(*t*_1_), …,*X*^*j*′^(*t*_*f*_)) are independent. Independence is usually denoted as $\left\{X_{t}^{j}\}⊥⊥\{X_{t}^{\;j^{\prime }}\right\}$. Below, we will abuse notation and use the shorthand $X_{t}^{j}⊥⊥X_{t}^{\;j^{\prime }}$.

From this definition, it immediately follows that the realizations are independent.

**Remark (Independence of realizations).**

Although the samples within a path $\left(X^{j}(1,\omega_{i}),\ldots ,X^{j}(T_{j},\omega_{i}))=(x_{i,1}^{j},\ldots ,x_{i,T_{j}}^{j}\right)$ are not necessarily independent across time, each variable is independent across realizations for any time *t*, i.e. $x_{i,t}^{j}⊥⊥x_{i^{\prime },t}^{j}\;\forall t,\;\forall i\neq i^{\prime }$. In other words, the *n* time series are assumed to be *iid* samples, ${\left\{x_{i}^{j}\right\}}_{i=1}^{n}\overset{iid}{∼}P_{X^{j}}$, where $P_{X^{j}}$ is a finite-dimensional distribution of the stochastic process $\left\{X_{t}^{j}\right\}$.

**Definition (Stationarity).**

A stochastic process is said to be stationary if all its finite-dimensional distributions are invariant under translations of time.

*Aim of the paper:* Here, we use kernels to embed finite-dimensional distributions of the *d* stochastic processes $\left\{X_{t}^{j}\right\}$ and design tests for joint independence of time series thereof. Recent work has used HSIC to test for independence of pairs of stationary [27,34] and non-stationary [28] time series. Here, we extend this work to *d* > 2 time series using tests based on dHSIC. We consider two scenarios:

— if we only observe a single time series (*n* = 1) of each of the *d* variables, then we can only consider stationary processes;
— if we have access to several time series (*n* > 1) of each of the *d* variables, then we can also study non-stationary processes.

## 3. dHSIC for joint independence of stationary time series

We first consider the scenario where we only have one time series (*n* = 1) for each of the *d* variables *X*^*j*^, which are all assumed to be stationary. Our dataset is then ${\left\{x^{j}\right\}}_{\;j=1}^{d}$, and it consists of *d* time-series vectors $x^{j}=(x_{1}^{j},\ldots ,x_{T}^{j})$, which we view as single realizations of the stationary stochastic processes $\left\{X_{t}^{\;j}\right\}$, all sampled at times *t* = 1, …,*T*. As will become clear below, the limited information provided by the single realization, together with the use of permutation-based statistical tests, means that the assumption of stationarity is necessary [26].

Let $K^{j}\in R^{T\times T}$ be kernel matrices with entries $K_{ab}^{j}=k^{j}(x_{a}^{j},x_{b}^{j})$ where *a*,*b* ∈ {1, …,*T*}, and $k^{j}\;:\;R\times R\to R$ is a characteristic kernel (e.g. Gaussian); hence, the matrix *K*^*j*^ captures the autocorrelational structure of variable *X*^*j*^. In this case, dHSIC (2.1) can be estimated as the following expansion in terms of kernel matrices [19,35]:

3.1 $${\hat{\mathrm{dHSIC}}}_{st}(x^{1},\ldots ,x^{d})\;:=\frac{1}{T^{2}}\sum_{a=1}^{T}\sum_{b=1}^{T}\prod_{\;j=1}^{d}K_{ab}^{j}-\frac{2}{T^{d+1}}\sum_{a=1}^{T}\prod_{\;j=1}^{d}\sum_{b=1}^{T}K_{ab}^{j}+\frac{1}{T^{2d}}\prod_{\;j=1}^{d}\sum_{a=1}^{T}\sum_{b=1}^{T}K_{ab}^{j}.$$

The null hypothesis is $H_{0}\;:\;P_{X^{1},\ldots ,X^{d}}=P_{X^{1}}\cdots P_{X^{d}}$, and we test (3.1) for statistical significance. To do so, we bootstrap the distribution under *H*_0_ using random shifting to generate *S* samples [26]. For each of the samples *s* = 1, …,*S*, we fix one time series (**x**^1^ without loss of generality) and generate random shifting points $\tau_{s}^{j},\;j=2,\ldots ,d$ for each of the other *d* − 1 time series where $h<\tau_{s}^{j}<T$ and *h* is chosen to be the first index where the autocorrelation of $\sum_{\;j=1}^{d}x^{j}$ is less than 0.2 [26].

**Figure 1.**
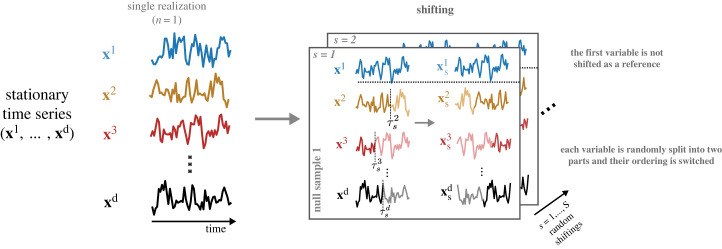
Shifting strategy for random sampling of single-realization stationary time series. The shifting method is used for stationary time series when only one realization of each variable is available. For each null sample *s*, the first time series **x**^1^ is kept fixed and a random shifting point $\tau_{j}^{s}$ is chosen for each of the other time series **x**^*j*^, *j* = 2, …, *d* so that the sections before and after $\tau_{j}^{s}$ (darker and lighter shades of colour) are switched. This process generates *S* randomly shifted samples that are used to bootstrap the null distribution.

Each time series is then shifted by $\tau_{s}^{j}$, so that $x_{s,t}^{j}=x_{(t+\tau_{s}^{j})\mathrm{mod}T}^{j}$. This shifting procedure, which is illustrated in figure 1, breaks the dependence across time series yet retains the local temporal dependence within each time series. In this way, we produce *S* randomly shifted datasets $(x_{s}^{1},\ldots ,x_{s}^{d}),\;s=1,\ldots ,S$, and the estimated dHSIC is computed for each shifting: ${\hat{\mathrm{dHSIC}}}_{st}(x_{s}^{1},\ldots ,x_{s}^{d})$. The p-value is computed by Monte Carlo approximation [19]. Given a significance level *α*, the null hypothesis *H*_0_ is rejected if α>p-value. We note that although an alternative to shifting called Wild Bootstrap has been proposed [27,36], it has been reported to produce large false positive rates [37]. We therefore use shifting (and not the Wild Bootstrap) in this manuscript.

### 3.1. Numerical results

#### 3.1.1. Validation on synthetic stationary multivariate systems with a single realization

To validate our approach, we apply the dHSIC test for joint independence to datasets consisting of *d* = 3 time series of length *T* with *n* = 1 realizations (i.e. one time series per variable). We use three stationary models with a known dependence structure (ground truth), the strength of which can be varied. For each test, we use *S* = 1000 randomly shifted samples and we take *α* = 0.05 as the significance level. We then generate 200 such datasets for every model and combination of parameters (*T*, *λ*), and compute either the test power (i.e. the probability that the test correctly rejects the null hypothesis when there is dependence) or the type I error (i.e. the probability that the test mistakenly rejects the true null hypothesis when there is independence) for the 200 datasets.

**Figure 2.**
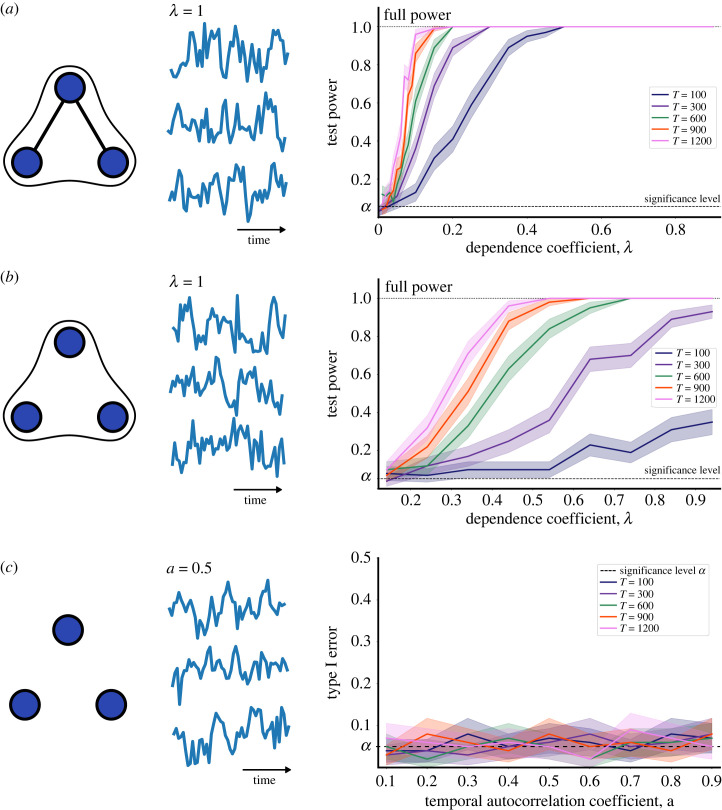
Stationary systems with a single realization. Left: a visualization of the ground truth dependencies where edges represent rejection of pairwise dependence and three-way hyperedges represent the rejection of three-way joint independence. Middle: an example of the three-variable time series for which dHSIC was computed. Right: test power (for *a*,*b*) and type I error (for *c*) computed by applying the dHSIC test to 200 datasets generated for each model with different dependence coefficient *λ* (autocorrelation coefficient *a* for *c*) and length of time series *T*. The lines represent the average over the 200 datasets and the shaded areas correspond to confidence intervals. The systems are taken from [38]: (*a*) three-way dependence ensuing from pairwise dependencies (3.2); (*b*) emergent three-way dependence with no underlying pairwise dependencies (3.3); and (*c*) joint independence (3.4).

*Model 1.1: Three-way dependence ensuing from pairwise dependencies.* The first stationary example [38] has a three-way dependence that follows from the presence of two simultaneous two-way dependencies:

3.2 $$X_{t}=\frac{1}{2}X_{t-1}+\epsilon_{t},\;Y_{t}=\frac{1}{2}Y_{t-1}+\eta_{t},\;Z_{t}=\frac{1}{2}Z_{t-1}+\zeta_{t}+\lambda (X_{t}+Y_{t}),$$

where $\epsilon_{t}$, *η*_*t*_, *ζ*_*t*_ and *θ*_*t*_ are generated as *iid* samples from a normal distribution N(0,1), and the dependence coefficient *λ* regulates the magnitude of the dependence between variables, i.e. for *λ* = 0 we have joint independence of (*X*, *Y*, *Z*) and the dependence grows as *λ* is increased. Figure 2*a* shows the result of our test for *d* = 3 variables applied to time series of length *T* = [100, 300, 600, 900, 1200] and increasing values of the dependence coefficient 0 ≤ *λ* ≤ 1 generated from model (3.3). As either *λ* or *T* increase, it becomes easier to reject the null hypothesis of joint independence. Full test power can already be reached for *λ* = 0.5 across all lengths of time series. Our test also rejects pairwise independence between the (*X*, *Z*) and (*Y*, *Z*) pairs, and fails to reject independence between (*X*, *Y*), as expected from the ground truth.

*Model 1.2: Pure three-way dependence.* Our second stationary example, also from [38], includes a three-way dependence without any underlying pairwise dependence:

3.3 $$X_{t}=\frac{1}{2}X_{t-1}+\epsilon_{t},\;Y_{t}=\frac{1}{2}Y_{t-1}+\eta_{t},\;Z_{t}=\frac{1}{2}Z_{t-1}+\zeta_{t}+\lambda |\theta_{t}|\text{sign}(X_{t}Y_{t}),$$

where $\epsilon_{t},\eta_{t},\zeta_{t}$ and *θ*_*t*_ are *iid* samples from N(0,1), and the coefficient *λ* regulates the three-way dependence. Figure 2*b* shows that the test rejects the null hypothesis as either *λ* or *T* increase, although the test power is lower relative to (3.2), as there are no two-way dependencies present in this case, i.e. this is a three-way emergent dependency.

*Model 1.3: Joint independence.* As a final validation, we use a jointly independent example [38]:

3.4 $$X_{t}=aX_{t-1}+\epsilon_{t},\;Y_{t}=aY_{t-1}+\eta_{t},\;Z_{t}=aZ_{t-1}+\zeta_{t},$$

where $\epsilon_{t}$, *η*_*t*_ and *ζ*_*t*_ are *iid* samples from N(0,1). Figure 2*c* shows that in this case we do not reject the null hypothesis of joint independence across a range of values of the autocorrelation parameter *a*. Note that the type I error of the test remains controlled around the significance *α* = 0.05 for all values of *T* and *a*.

#### 3.1.2. Synthetic frequency mixing data

**Figure 3.**
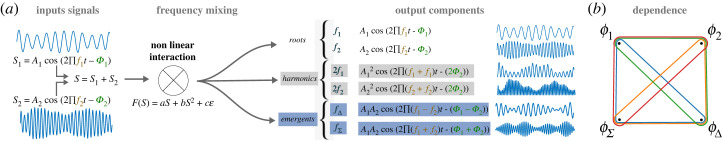
Frequency mixing. (*a*) Two independent input signals with root frequencies *f*_1_ =7 Hz and *f*_2_ =18 Hz are mixed via a quadratic function with noise to generate the signal *F*. This signal has components at the root frequencies, harmonics (double of the root frequencies) and emergents (sum and difference of the root frequencies), as shown by the output waveforms (right). Time series of the instantaneous phases $\phi_{1},\phi_{2},\phi_{\Delta },\phi_{\Sigma }$ are extracted from the output components at $f_{1},f_{2},f_{\Delta },f_{\Sigma }$ and dHSIC is applied to them. (*b*) In this case, the dHSIC test does not reject pairwise independence between any pair of variables (i.e. there are no pairwise dependencies), but rejects the joint independence between any three of the four variables (i.e. four three-way emergent dependencies are present), as shown by the triangles, and consequently also rejects the joint independence between the four variables (square).

As a further illustration linked more closely to real-world applications, we have generated a dataset based on frequency mixing of temporal signals. Frequency mixing is a well-known phenomenon in electrical engineering, widely used for heterodyning, i.e. shifting signals from one frequency range to another. Applying a nonlinear function (e.g. a quadratic function or a rectifier) to the sum of two signals with distinct frequencies generates new signals with emergent frequencies at the sum and difference of the input signals (figure 3*a*–*c*). It has previously been shown that the instantaneous phases of the emergents display a unique three-way dependence, without any pairwise dependencies [39–41]. Importantly, given sufficiently long time series, the instantaneous phase can be considered a stationary signal [39]. Hence, we can apply our test to this system.

Here, we generated a dataset using the sum of two sinusoidal functions with frequencies *f*_1_ = 7 Hz and *f*_2_ = 18 Hz as input, to which we applied a quadratic function plus weighted Gaussian noise ϵ. This produces a signal *F* that contains components at input (root) frequencies (*f*_1_ = 7 Hz and *f*_2_ = 18 Hz), second harmonics (2*f*_1_ = 14 Hz and 2*f*_2_ = 36 Hz) and emergent frequencies ($f_{\Delta }=f_{2}-f_{1}=11\;\mathrm{Hz}$ and $f_{\Sigma }=f_{1}+f_{2}=25\;\mathrm{Hz}$). See figure 3*a* and [39] for further details. We then computed a wavelet transform and extracted the instantaneous phases for frequencies $f_{1},f_{2},f_{\Sigma }$ and $f_{\Delta }$, which we denoted $\phi_{1},\phi_{2},\phi_{\Sigma }$ and $\phi_{\Delta }$. These phases can be considered as stationary time series. The ground truth is that there should be no pairwise dependencies between any of those phases, but there are higher-order interactions involving three-way and four-way dependencies [39].

We applied dHSIC with shifting to all possible groupings of *d* phases (for *d* = 2, 3, 4) from the set $\left\{\phi_{1},\phi_{2},\phi_{\Sigma },\phi_{\Delta }\right\}$. The phases consisted of time series with length *T* = 1000, and we used *S* = 1000 shiftings for our bootstrap. We found that the null hypothesis of independence could not be rejected for any of the six phase pairs (*d* = 2), whereas joint independence was rejected for all four phase triplets (*d* = 3) and for the phase quadruplet (*d* = 4). The rejection of all the three-way and four-way joint independence hypotheses, without rejection of any of the pairwise independence hypotheses, thus recovers the ground truth expected structure (figure 3*b*).

#### 3.1.3. Application to climate data

As an application to real-world data, we used the PM2.5 air quality dataset, which contains four variables: hourly measurements of particulate matter with a diameter of 2.5 microns or less (PM2.5) recorded by the US Embassy in Beijing between 2010 and 2014, and three concurrent meteorological variables (dew point, temperature, air pressure) measured at Beijing Capital International Airport [42]. Non-stationary trends and yearly seasonal effects were removed by taking differences of period 1 and period 52 in the averaged weekly data. Stationarity of the de-trended series was verified by an Adfuller test [43]. As expected, we found that the null hypotheses (joint independence) were rejected for all groups of *d* = 2, 3, 4 variables, implying that PM2.5, dew point, temperature and air pressure are all dependent on each other.

## 4. dHSIC for joint independence of non-stationary time series with multiple realizations

When we have multiple independent observations of the *d* variables, these can be viewed as *iid* samples of a multivariate probability distribution. By doing so, the requirements of stationarity and same point-in-time measurements across all variables can be loosened.

Consider the case when we have access to *n* > 1 observations of the set of variables (*X*^1^, …, *X*^*d*^), where each observation *i* = 1, …, *n* consists of *d* time series *X*^*j*^, which we write as vectors $x_{i}^{j}=(x_{i,1}^{j},\ldots ,x_{i,T_{j}}^{j})$ of length *T*_*j*_. Each of the *n* observations thus consists of a set ${\left\{x_{i}^{j}\right\}}_{\;j=1}^{d}$, which can be viewed as an independent (*iid*) realization of a finite-dimensional multivariate distribution $P_{X^{1},\ldots ,X^{d}}$. To simplify our notation, we compile the *n* observations of each *X*^*j*^ as rows of a *n* × *T*_*j*_ matrix **X**^*j*^, so that $X_{\left[i,\;:\;\right]}^{j}=x_{i}^{j}$.

Let $\kappa^{j}\;:\;R^{T_{j}}\times R^{T_{j}}\to R$ be a characteristic kernel (e.g. Gaussian) that captures the similarity between a pair of time series of variable *X*^*j*^. We then define the set of kernel matrices $K^{j}\in R^{n\times n}$ with entries $K_{\alpha \beta }^{j}=\kappa^{j}(x_{\alpha }^{j},x_{\beta }^{j})$ where *α*, *β* ∈ {1, …, *n*}. Therefore, the matrix $K^{j}$ captures the similarity structure between the time series of variable *X*^*j*^ across the *n* observations. This set-up thus allows us not to require stationarity in our variables, since the *n* observations capture the temporal behaviour of the *d* variables concurrently. In this case, dHSIC for the set of observations (**X**^1^, …, **X**^*d*^) can be estimated as [19]

4.1 $${\hat{\mathrm{dHSIC}}}_{\mathrm{mult}}(X^{1},\ldots ,X^{d})\;:=\frac{1}{n^{2}}\sum_{\alpha =1}^{n}\sum_{\beta =1}^{n}\prod_{\;j=1}^{d}K_{\alpha \beta }^{j}-\frac{2}{n^{d+1}}\sum_{\alpha =1}^{n}\prod_{\;j=1}^{d}\sum_{\beta =1}^{n}K_{\alpha \beta }^{j}+\frac{1}{n^{2d}}\prod_{\;j=1}^{d}\sum_{\alpha =1}^{n}\sum_{\beta =1}^{n}K_{\alpha \beta }^{j}.$$

**Figure 4.**
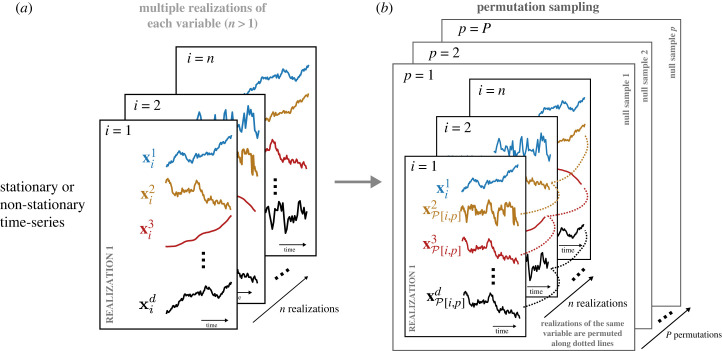
Random permutation sampling of multivariate time series with multiple realizations. A permutation strategy similar to the one developed for *iid* data [19] can be applied when multiple realizations of either stationary or non-stationary time-series data are available. Each null sample *p* = 1, …, *P* is generated by randomly permuting the time series for variables *j* = 2, …, *d* across realizations i=1,…,n, as indicated by the dotted lines, while the first variable remains unchanged. Null distributions are generated from independent samples of this process.

Similarly to §3, the null hypothesis is $H_{0}\;:\;P_{X^{1},\ldots ,X^{d}}=P_{X^{1}}\cdots P_{X^{d}}$ and we test (4.1) for statistical significance. Due to the availability of multiple realizations, however, we use a different resampling method (standard permutation test) to bootstrap the distribution of (4.1) under *H*_0_ (figure 4). For each of the samples *p* = 1, …, *P*, we fix one variable (*X*^1^ without loss of generality), and we randomly permute the rest of the variables *across realizations* to create the permuted sample ${\left\{x_{p}^{j}\right\}}_{\;j=1}^{d}={\left\{x_{P[i,p]}^{j}\right\}}_{\;j=1}^{d}$, where P[i,p] indicates a random permutation between realizations, and $x_{p}^{1}=x_{i}^{1},\;\forall p$. In this way, we produce *P* permuted datasets $(X_{p}^{1},\ldots ,X_{p}^{d}),\;p=1,\ldots ,P$, with $X_{p}^{1}=X^{1}$. The estimated dHSIC (4.1) is then computed for each permutation *p*. Given a significance level *α*, the null hypothesis *H*_0_ is rejected if α>p−value where the p-value is computed by Monte Carlo approximation [19].

### 4.1. Numerical results

#### 4.1.1. Validation on simple non-stationary multivariate systems

**Figure 5.**
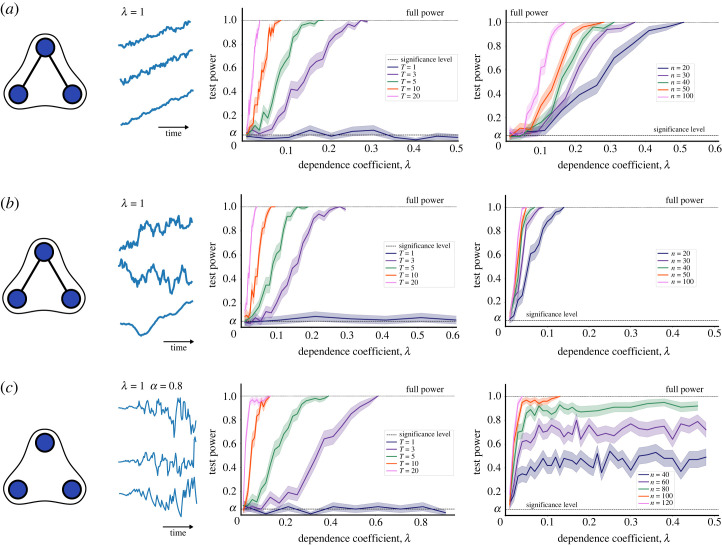
Three-variable non-stationary systems with multiple realizations. Left: a visualization of the ground truth dependencies, where edges represent pairwise dependence and 3-edges represent a three-way dependence. Middle left: an example of a realization of a three-variable time series. Middle right: test power computed by applying the dHSIC test to 200 datasets generated from a model at varying values of the dependence coefficient *λ* and the length of time series *T*, with a fixed number of realizations *n* = 100. Right: test power computed by applying the dHSIC test to 200 datasets generated from a model at varying values of the dependence coefficient *λ* and the number of realizations *n*, with a fixed length of time series *T* = 20. The lines represent the average over the 200 datasets and the shaded areas correspond to confidence intervals. The systems are: (*a*,*b*) three-way dependence ensuing from two-way dependencies (4.2): (*a*) linear trend, (*b*) a complex dependence term; and (*c*) three-way dependence with no underlying pairwise dependencies and a non-stationary trend (4.3).

The dHSIC test is applied to datasets consisting of *n* observations of non-stationary time series of length *T* of three variables (*X*, *Y*, *Z*), with ground truth dependencies that can be made stronger by increasing a dependence coefficient *λ*. For every model and combination of parameters (*n*, *T*, *λ*), we generate 200 datasets and compute the test power, i.e. the probability that the test correctly rejects the null hypothesis in our 200 datasets. Figure 5 shows our numerical results for two non-stationary models: the first model (shown in figure 5*a*,*b* with two non-stationary trends) has a three-way dependence ensuing from two-way dependencies; the second model (shown in figure 5*d* for a non-stationary trend) has an emergent three-way dependence with no pairwise dependencies.

*Model 2.1: Three-way dependence ensuing from pairwise dependencies with non-stationarity.* The first model has the same dependence structure as (3.2), i.e. two simultaneous pairwise dependencies and an ensuing three-way dependence, but in this case with non-stationary trends:

4.2 $$X_{t}=g_{1}(t)+X_{t-1}+\epsilon_{t},\;Y_{t}=g_{2}(t)+Y_{t-1}+\eta_{t},\;Z_{t}=g_{3}(t)+Z_{t-1}+\zeta_{t}+\lambda (X_{t}+Y_{t}),$$

where $\epsilon_{t},\eta_{t}\;\mathrm{and}\;\zeta_{t}$ are *iid* samples from a normal distribution N(0,1); *λ* regulates the strength of the dependence (*λ* = 0 means joint independence); and *g*_1_(*t*), *g*_2_(*t*), *g*_3_(*t*) are non-stationary trends as follows:

— linear trend (figure 5*a*): *g*_1_(*t*) = *g*_2_(*t*) = *g*_3_(*t*) = *t*
— complex nonlinear trend (figure 5*b*): $g_{1}(t)=\sin^{2}(t)/\log(1+t)$, $g_{2}(t)=\cos^{2}(t)/\log(1+t)$, $g_{3}(t)=\sin(t)\cos(t)/\log(1+t)$.

Figure 5*a*,*b* shows that the dHSIC test is able to reject the null hypothesis of joint independence for (4.2) even for short time series and low values of the dependence coefficient *λ*. The test power increases rapidly as the length of the time series *T* or the number of realizations *n* are increased. As expected, the null hypothesis cannot be rejected for *T* = 1, since the temporal dependence is no longer observable.

*Model 2.2: Emergent three-way dependence with non-stationarity.* The second model has the same dependence structure as (3.4) (i.e. an emergent three-way dependence without two-way dependencies) but with non-stationary trends

4.3 $$X_{t}=aX_{t-1}+\epsilon_{t}+t\sin(t),\;Y_{t}=aY_{t-1}+\eta_{t}+t\cos(t),\;Z_{t}=aZ_{t-1}+\zeta_{t}+\lambda \;t\text{sign}(X_{t}Y_{t}),$$

where, again, $\epsilon_{t}$, *η*_*t*_ and *ζ*_*t*_ are *iid* samples from N(0,1), and *λ* regulates the strength of the dependence. We set *a* = 0.8, the point at which the data becomes non-stationary according to an Adfuller test. Figure 5*c* shows good performance of the test, which is able to reject joint independence for low values of *λ*, with increasing test power as the length of the time series *T* and the number of realizations *n* are increased (figure 5*c*).

#### 4.1.2. Synthetic XOR dependence

The exclusive OR (XOR) gate (denoted ⊕) is a logical device with two Boolean (0–1) inputs and one Boolean output, which returns a 1 when the number of ‘1’ inputs is odd. Here, we consider a system with three Boolean variables *X*, *Y*, *W* driven by noise, which get combined via XOR gates to generate another Boolean variable *Z*:

4.4 $$\begin{array}{cc} & \;X_{t}=\left\{ \begin{array}{cc} X_{t-1},\; & \text{if}\;\epsilon_{t}\geq 0.5\;\\ 1-X_{t-1},\; & \text{otherwise}\; \end{array}\right.\;Y_{t}=\left\{ \begin{array}{cc} Y_{t-1},\; & \text{if}\;\eta_{t}\geq 0.5\;\\ 1-Y_{t-1},\; & \text{otherwise}\; \end{array}\right.\\ \;\mathrm{and}\;\;\;\; & W_{t}=\left\{ \begin{array}{cc} W_{t-1},\; & \text{if}\;\zeta_{t}\geq 0.5\;\\ 1-W_{t-1},\; & \text{otherwise}\; \end{array}\right.\;Z_{t}=\left\{ \begin{array}{cc} X_{t}⊕Y_{t}⊕W_{t},\; & \text{if }\theta_{t}\geq 0.5\;\\ 1-X_{t}⊕Y_{t}⊕W_{t},\; & \text{otherwise}\; \end{array}\right. \end{array}\}\;\;\;$$

where $\epsilon_{t}$, *η*_*t*_, *ζ*_*t*_ and *θ*_*t*_ are *iid* samples from U[0,1), a uniform distribution between 0 and 1, and *X*_0_, *Y*_0_ and *W*_0_ are initialized as random Boolean variables. The dependence in this system is high-order: it only appears when considering the four variables, with no three-way or two-way dependencies. We find that our test does not reject joint independence for *d* = [2, 3] variables, but does reject joint independence of the four-variable case.

#### 4.1.3. Application to MRI and Alzheimer’s data

As a first application to data with multiple realizations, we apply our test to a magnetic resonance imaging (MRI) and Alzheimer’s longitudinal dataset [44], which comprises demographic and MRI data collected from subjects over several visits. Here, we consider *n* = 56 subjects, each with at least three visits (*T* = 3), and we assume that the subjects constitute *iid* realizations—a reasonable assumption since this is a well-designed population study with representative samples. We then perform dHSIC tests to find dependencies between four key variables: age, normalized whole brain volume (nWBV), estimated total intracranial volume (eTIV) and clinical dementia rating (CDR). The first three variables are clinical risk factors, whereas CDR is a standardized measure of disease progression.

**Figure 6.**
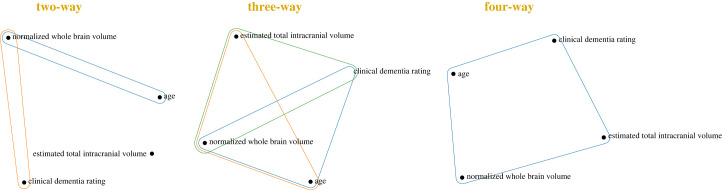
High-order dependencies between four variables in MRI and Alzheimer’s data containing multiple realizations of time-series data. The hyperedges represent rejections of the respective joint independence tests. We find 2 (out of 6) pairwise dependencies and 3 (out of 4) three-way dependencies, as well as the four-way dependence between all variables. There are no emergent dependencies in this example.

Our findings are displayed as hypergraphs in figure 6 where nodes represent variables and hyperedges represent rejections of joint independence from the two-way, three-way and four-way dHSIC tests. In this case, we find only two pairwise dependencies (age-nWBV and nWBV-CDR), while eTIV is seemingly disconnected to the rest of the variables. Note that the possible emergent three-way interaction (age-eTIV-CDR) is not present, although eTIV shows the expected three-way and four-way dependencies with CDR, nWBV and age. This example highlights how our method can be used to reveal the different higher-order dependencies beyond pairwise interactions. To understand the complex high-order interactions of the incomplete factorizations, methods based on Streitberg and Lancaster interaction can be explored in future work [45].

#### 4.1.4. Application to socio-economic data

As a final illustration in a different domain area, we test for joint independence between the United Nations SDGs [46]. This dataset consists of a time series of a large number of socio-economic indicators forming the 17 SDGs (our variables *X*^*j*^, *j* = 1, …, 17) measured yearly between 2000 and 2019 (*T* = 20) for all 186 countries in the world (see [12] for details on the dataset). We take the countries to be *iid* realizations, as in [12], although this assumption is less warranted here than for the dementia dataset in §4.1.3 due to moderate correlations between countries due to socio-economic and political relationships.

As an illustration of the differences in data dependencies across country groupings, we consider two classic splits: (i) a split based on income level (*n* = 74 countries with low and lower-middle income, and *n* = 105 countries with high and upper-middle income); and (ii) a split based on broad geography and socio-economic development (*n* = 49 countries in the Global North and *n* = 137 countries in the Global South). This dataset highlights the difficulties of examining high-order dependencies as the number of variables grows, e.g. *d* = 17 in this case.

**Figure 7.**
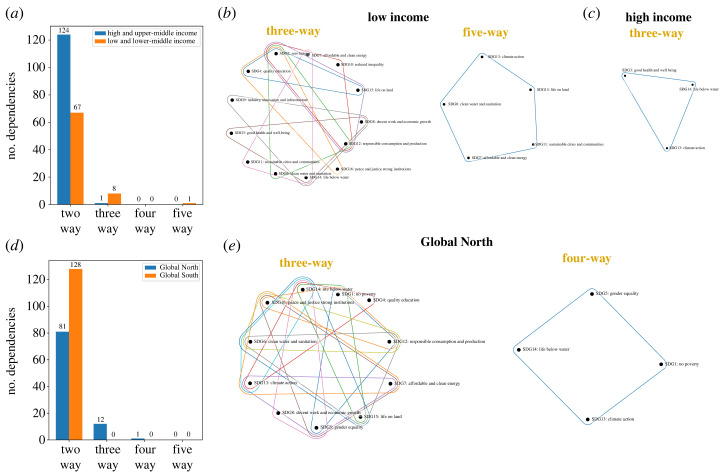
Emergent high-order dependencies between SDGs. (*a*–*c*) Comparison of SDG dependencies in low- and high-income countries. (*a*) There is a higher number of emergent higher-order dependencies in low-income countries. The *d* > 2 dependencies are mapped onto *d*-order hypergraphs for (*b*) low-income and (*c*) high-income countries. (*d*,*e*) Comparison of SDG dependencies in Global North and Global South countries. (*d*) Emergent high-order dependencies are found in the Global North, whereas the Global South displays only two-way dependencies. (*e*) The *d* > 2 dependencies for the Global North are mapped onto *d*-order hypergraphs.

The results of applying this recursive scheme to the SDG dataset are shown in figure 7. The comparison between low- and high-income countries (figure 7*a*–*c*) shows that the latter have strong pairwise dependencies (124 rejections of two-way independence out of a total of 136 pairs) and only 1 emergent three-way interaction (figure 7*c*), whereas the former have more emergent higher-order dependencies (eight three-way and one five-way) (figure 7*b*). These results suggest that the interdependencies between SDGs are more complex for lower-income countries, whereas most of the high-order dependencies in high-income countries are explained by the pairwise dependencies between indicators. Given that many analyses of SDG interlinkages consider only pairwise relationships, this implies the need to consider high-order interactions to capture relationships in lower-income countries where policy actions targeting pairwise interlinkages could be less effective. The comparison between the Global North and Global South (figure 7*d*,*e*) shows that the latter has exclusively two-way dependencies, whereas the former has emergent three-way interactions (12) and four-way interactions (1) (figure 7*e*). Interestingly, two SDGs, climate action and life below water, consistently appear in emergent high-order dependencies in lower- and higher-income countries, and in Global North groupings, suggesting their potential for further studies. In addition, the hypergraphs of emergent high-order interactions for different country groupings can be studied using network science techniques, including the computation of centrality measures to rank the importance of SDGs within the system of interdependent SDG objectives and the use of community detection algorithms to extract clusters of highly interdependent SDGs [12].

## 5. Discussion

In this paper, we present dHSIC tests for joint independence in both stationary and non-stationary time-series data. For single realizations of stationary time series, we employ a random shifting method as a resampling technique. In the case of multiple realizations of either stationary or non-stationary time series, we consider each realization as an independent sample from a multivariate probability distribution, enabling us to use random permutation as a resampling strategy. To validate our approach, we conducted experiments on diverse synthetic examples, successfully recovering ground truth relationships, including in the presence of a variety of non-stationary behaviours. As illustrated by applications to climate, SDGS, and MRI and Alzheimer’s data, the testing framework could be applicable to diverse scientific areas in which stationary or non-stationary time series are the norm.

**Figure 8.**
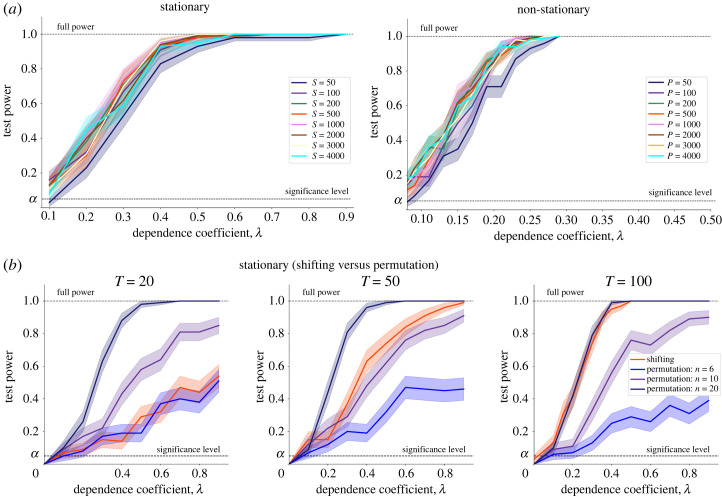
Robustness and efficiency of shifting and permutation resampling strategies. (*a*) Test power for the same model in its stationary version (Model 1.1 (3.2), using shifting resampling, left) and non-stationary version (Model 2.1 (4.2) with linear trend, using permutation resampling, right). Relatively few null samples (*S*, *P* > 100) are enough to attain high test power for both schemes. (*b*) For stationary time series with multiple realizations, both shifting and permutation can be employed. Shifting is preferred if the number of time observations (*T*) is large relative to the number of realizations (*n*); conversely, permutation is preferred if *n* is large relative to *T*. For Model 1.1 (3.2) with *T* = 20, the permutation scheme with *n* = 6 already reaches comparable performance to shifting, whereas for *T* = 100 we need *n* = 20 for permutation resampling to reach comparable performance to shifting. We use *S* = *P* = 1000 for all tests in (*b*).

There are some computational considerations that need to be taken into account for different applications. In our numerical experiments, we have evaluated the impact of several parameters, including the length of the time series *T* and the number of observations *n*, on the computational efficiency and statistical power of our test. In general, the test statistic can be computed in $O(dT^{2})$ or $O(dn^{2})$, where *d* is the number of variables and *T*^2^ or *n*^2^ are the sizes of the kernel matrices [19]. Hence, the computational cost increases with the number of variables and/or number of realizations and length of the time series. The computational cost also grows linearly with the number of resamplings (*S* or *P*) used to approximate the null distribution, but our findings show that the test is robust even for low numbers of resamplings. Figure 8*a* shows that the test power does not improve substantially beyond 100 resamplings (permutations)—a result that has been previously discussed for *iid* data [47]. Therefore, achieving a balance between test power and computational efficiency is crucial, particularly when dealing with large multivariate datasets.

It is worth noting that for stationary data with multiple independent realizations, both resampling schemes (shifting and permutation) can be employed to sample the null distribution. If the number of realizations (*n*) is much larger than the length of the time series (*T*), the permutation strategy provides more efficient randomization as long as the realizations are diverse. Conversely, when *n* is smaller than *T*, time shifting allows to better exploit the observed temporal dynamics. As an illustration of this point for Model 1.1 (3.2) with multiple realizations, figure 8*b* shows that if we have *T* = 20 time points available, then the permutation-based approach has the same performance as the shifting approach when the number of realizations reaches *n* = 6. However, if *T* = 100 time points are available, both performances become similar when the number of realizations is *n* = 20. These resampling alternatives must also be evaluated in conjunction with the study of different kernels that can more effectively capture the temporal structure within and across time series (e.g. signature kernels). We leave the investigation of these areas as an avenue of future research.

The interest in higher-order networks, such as hypergraphs or simplicial complexes, has been steadily growing [24] with applications across scientific fields [22,48–51]. Higher-order networks can be natural formalizations of relational data linking *d* entities [52,53]. However, there is a scarcity of research and a lack of consensus on how to *construct* higher-order networks from observed *iid* or time-series data [54], and the joint independence methods proposed here could serve to complement approaches based on information measures [20]. By iteratively testing from pairwise independence up to *d*-order joint independence, our approach can uncover emergent dependencies not explained by lower-order relationships. This framework presents a direction for the development of higher-order networks, bridging the gap between observed data and the construction of meaningful higher-order network representations.

## Data Availability

Climate data: http://dx.doi.org/10.24432/C5JS49, SDG data: https://datacatalog.worldbank.org/dataset/sustainable-development-goals, and MRI and Alzheimer’s data: https://www.kaggle.com/datasets/jboysen/mri-and-alzheimers. Synthetic examples: the code to generate the synthetic data and the algorithms to implement the tests are available at https://github.com/barahona-research-group/dHSIC_ts.
